# A Recombinant Influenza A/H1N1 Carrying A Short Immunogenic Peptide of MERS-CoV as Bivalent Vaccine in BALB/c Mice

**DOI:** 10.3390/pathogens8040281

**Published:** 2019-12-02

**Authors:** Mahmoud M. Shehata, Ahmed Kandeil, Ahmed Mostafa, Sara H. Mahmoud, Mokhtar R. Gomaa, Rabeh El-Shesheny, Richard Webby, Ghazi Kayali, Mohamed A. Ali

**Affiliations:** 1 Center of Scientific Excellence for Influenza Viruses, National Research Centre (NRC), Dokki, Cairo 12622, Egypt; Mahmoud.Shehata@human-link.org (M.M.S.); ahmed.Kandeil@human-link.org (A.K.); ahmed_elsayed@daad-alumni.de (A.M.); sarahussein9@yahoo.com (S.H.M.); Mokhtar.Rizk@human-link.org (M.R.G.); rabeh.elshesheny@stjude.org (R.E.-S.); 2 Department of Infectious Diseases, St. Jude Children’s Research Hospital, Memphis, TN 38105, USA; richard.webby@stjude.org; 3 Department of Epidemiology, Human Genetics, and Environmental Sciences, University of Texas, Houston, TX 77030, USA; 4 Human Link, Baabda 1109, Lebanon

**Keywords:** influenza vaccine, MERS-CoV, H1N1pdm, reverse genetics

## Abstract

Middle East Respiratory Syndrome Coronavirus (MERS-CoV) became a global human health threat since its first documentation in humans in 2012. An efficient vaccine for the prophylaxis of humans in hotspots of the infection (e.g., Saudi Arabia) is necessary but no commercial vaccines are yet approved. In this study, a chimeric DNA construct was designed to encode an influenza A/H1N1 NA protein which is flanking immunogenic amino acids (aa) 736–761 of MERS-CoV spike protein. Using the generated chimeric construct, a novel recombinant vaccine strain against pandemic influenza A virus (H1N1pdm09) and MERS-CoV was generated (chimeric bivalent 5 + 3). The chimeric bivalent 5 + 3 vaccine strain comprises a recombinant PR8-based vaccine, expressing the PB1, HA, and chimeric NA of pandemic 2009 H1N1. Interestingly, an increase in replication efficiency of the generated vaccine strain was observed when compared to the PR8-based 5 + 3 H1N1pdm09 vaccine strain that lacks the MERS-CoV spike peptide insert. In BALB/c mice, the inactivated chimeric bivalent vaccine induced potent and specific neutralizing antibodies against MERS-CoV and H1N1pdm09. This novel approach succeeded in developing a recombinant influenza virus with potential use as a bivalent vaccine against H1N1pdm09 and MERS-CoV. This approach provides a basis for the future development of chimeric influenza-based vaccines against MERS-CoV and other viruses.

## 1. Introduction

A novel human coronavirus, Middle East Respiratory Syndrome Coronavirus (MERS-CoV), was first detected in September 2012 in Saudi Arabia in a patient showing fever, shortness of breath, cough, and expectoration [1]. Since the first case, MERS-CoV spread rapidly around the world and 2428 human infections with MERS-CoV including 838 deaths were reported in four continents (Asia, Europe, Africa, and North America) as of July 2019 according to WHO. Most cases were reported in countries of the Arabian Peninsula, especially Saudi Arabia [2].

Using self-limited viruses as vectors for the immunogenic proteins of MERS-CoV has been applied in many studies. For instance, one study has engineered a recombinant human adenoviral vector to encoding the full S protein or the S1 domain protein of HCoV-EMC/2012 isolate. Both adeno-based vaccines were evaluated in BALB/c mice and found to induce T-cell responses and neutralizing antibodies (nAbs) against MERS-CoV [3,4,5]. Similarly, a recombinant modified vaccinia virus expressing the full S protein was developed. This vaccinia-based vaccine was evaluated against MERS-CoV in Ad5-hDPP4-transduced BALB/c mice and showed significant induction of humoral and cell-mediated immunity and nAbs [6,7]. In addition, using Ad5-hDPP4-transduced BALB/c mice immunized with Venezuelan equine encephalitis virus replicon particles containing S protein resulted in a reduction of viral titers to nearly undetectable levels by 1 day post infection (pi) and increased nAbs [8]. Vesicular stomatitis virus (VSV) expressing spike gene of MERS-CoV induced immunological T-cell response and virus neutralization in rhesus monkeys against VSV and MERS-CoV with a single immunization [9].

Influenza is a respiratory communicable disease caused by influenza viruses (IVs), which are classified antigenically based on the distinctions of the nucleoprotein (NP) into four genera: Influenza-A viruses (IAV), influenza-B viruses (IBV), influenza-C viruses (ICV), and influenza-D viruses (IDV) [10]. IVs have single-stranded, segmented, viral RNA genome (vRNA). Both IAV and IBV contain eight segments, while ICV and IDV contain only seven segments [11]. The genomic eight segments of IAV translate to more than 10 viral proteins [12]. 

Due to the well-studied safety profile of IAVs and their easy propagation and production using either embryonated chicken eggs or cell culture systems, several studies have used recombinant IAVs to either express certain immune modulators and anticancer proteins to combat cancer cells (oncolytic virus) [13,14]. 

Yang et al. (2014), synthesized short peptide 25 amino acid (aa) residues from spike protein of MERS-CoV at position 736–761 (CALPDTPSTLTPRSVRSVPGEMRLA), which elicited nAbs in mice and rabbits against MERS-CoV [15]. Herein, we used a recombinant influenza A H1N1pdm09pandemic (A/California/04/2009) as a PR8-based vector to express an immunogenic short peptide residue of the spike protein of MERS-CoV elicited immunity against MERS-CoV and H1N1pdm09. This short peptide was inserted in the neuraminidase gene of A/California/04/2009 (H1N1) for production of a PR8-based bivalent vaccine against influenza and MERS-CoV. 

## 2. Results

### 2.1. Generation of Recombinant Viruses by Reverse Genetics

**Figure 1:**
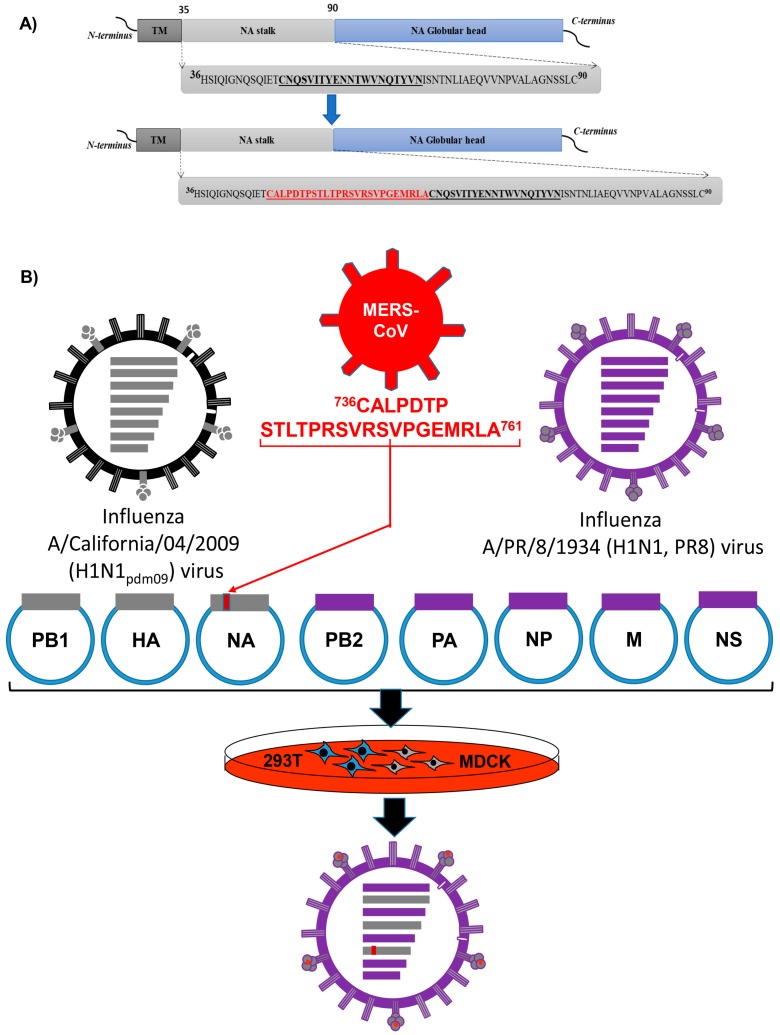
Schematic diagram of bivalent influenza vaccine strain. (**A**) Construction of recombinant neuraminidase (NA) gene by insertion of a short-peptide from Middle East Respiratory Syndrome Coronavirus (MERS-CoV)-Spike (red color) in NA stalk region of H1N1pdm2009 forming chimeric bivalent construct. Abbreviations: (TM) trans-membrane domain, (NA) neuraminidase. (**B**) Schematic diagram for the reverse genetics (rg) process to generate a candidate chimeric bivalent 5 + 3 virus carrying small peptide as a bivalent vaccine against H1N1pdm09 and MERS-CoV.

The short immunogenic peptide ^736^CALPDTPSTLTPRSVRSVPGEMRLA^761^ from MERS-CoV spike glycoprotein was inserted into the stalk region of NA gene of influenza A/California/04/2009 (H1N1) (H1N1pdm09) virus using the designed primers. A new recombinant H1N1 NA carrying short peptide of MERS-CoV (chimeric bivalent 5 + 3) was successfully constructed by recombinant ligation and cloning in pHW2000 vector (Figure 1A). Using the eight-plasmid reverse genetics system, recombinant 5 + 3 rgH1N1 (Control virus), and chimeric bivalent 5 + 3 (candidate bivalent virus) were generated. The reverse genetic system to generate the chimeric bivalent virus is illustrated in Figure 1B. The rescued bivalent and control viruses were propagated in SPF embryonated chicken eggs for three passages and were safe to embryos (no mortality) at 48 h post infection (pi). The inserted sequence for the immunogenic peptide of MERS-CoV spike glycoprotein was stable without any associated adaptive mutations/deletions in the NA gene during three successive passages in eggs. 

### 2.2. Growth Kinetic for the Novel Recombinant Virus

**Figure 2:**
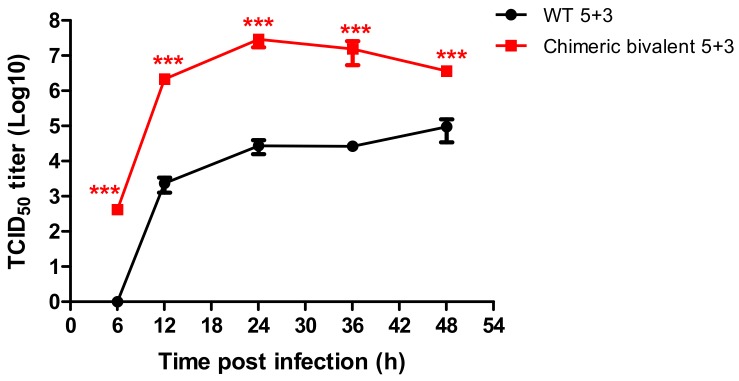
Growth kinetic curve of the new rescued candidate vaccine strain rgH1N1-MERS-CoV (chimeric bivalent 5 + 3) in comparison with the parent wild type rgH1N1 virus (WT 5 + 3) in MDCK-II cells at a multiplicity of infection (MOI) of 0.001. The experiments data represented for mean of three replicates ± standard error mean (SEM). Statistical analysis was performed using repeated measures ANOVA, followed by Bonferroni post hoc test. The significant differences are indicated (* = *p* < 0.05, ** = *p* < 0.01, *** = *p* < 0.001 and nonsignificant = ns).

The replication efficiency of the chimeric rgH1N1-MERS-CoV virus (chimeric bivalent 5 + 3) versus its parental counterpart without inserted MERS-CoV peptide (WT 5 + 3) was compared in MDCK-II cells. The chimeric virus had higher replication efficiency as measured by TCID_50_ at different time points, compared to the parental WT 5 + 3 virus (*p* < 0.05) (Figure 2). 

### 2.3. Evaluation of New Candidate Chimeric Bivalent Vaccine in BALB/c Mice

**Figure 3:**
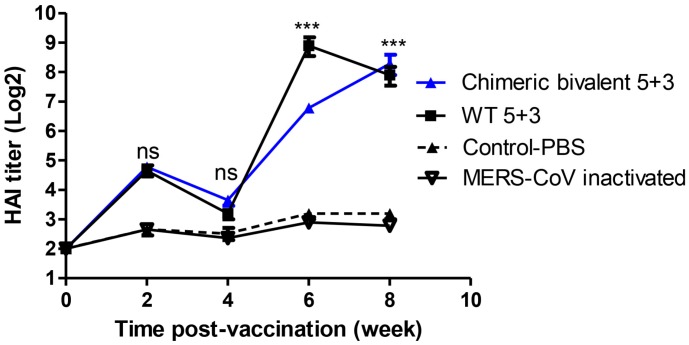
Weekly antibody hemagglutination inhibition hemagglutination inhibition (HAI) titers follow-up of vaccinated mice with chimeric bivalent 5 + 3 and WT 5 + 3 against rgH1N1 virus including control-phosphate buffer saline (PBS) group. The experiments data represented for mean of three replicates ± SEM (seven mice/group). Statistical analysis was performed using repeated measures ANOVA, followed by Bonferroni post hoc test. The significant differences are indicated (* = *p* < 0.05, ** = *p* < 0.01, *** = *p* < 0.001 and nonsignificant = ns).

Four groups of mice were intramuscularly injected with inactivated chimeric bivalent 5 + 3, WT 5 + 3, MERS-CoV vaccines, and 1× phosphate buffer saline (PBS). The collected sera of vaccinated groups with inactivated chimeric bivalent 5 + 3 and WT 5 + 3 revealed an increase in antibody titers at two weeks post vaccination (wpv). At week 4, the hemagglutination inhibition (HAI) titers waned after booster vaccination at week 3. The chimeric bivalent 5 + 3 vaccine showed a significant increase in mean HAI titer reaching 6.5 and 8.2 log_2_ at weeks 6 and 8, respectively, in comparison with control-PBS group. The inactivated WT 5 + 3 control group showed statistically significant increase in HAI titer at weeks 6 and 8 in comparison with control-PBS and MERS-CoV inactivated groups. These results revealed that the vaccinated mice have a potent immunogenic response against rgH1N1 virus as shown in Figure 3. 

**Figure 4:**
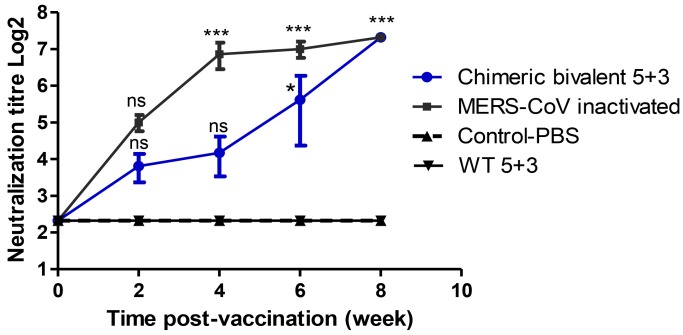
Neutralization antibodies titer against MERS-CoV in mice sera vaccinated with inactivated chimeric bivalent 5 + 3 and MERS-CoV viruses. Plaque reduction neutralization test 50 (PRNT_50_) was used for measuring antibodies titers in mice sera in Vero-E6 cells. The experimental data represented by mean of three replicates ± SEM (seven mice/group). Statistical analysis was performed using repeated measures ANOVA, followed by Bonferroni post hoc test. The significant differences are indicated (* = *p* < 0.05, ** = *p* < 0.01, *** = *p* < 0.001 and nonsignificant = ns).

Plaque reduction neutralization test (PRNT_50_) showed a high significant neutralizing titer in inactivated MERS-CoV vaccinated mice 1:160 titer (7.3 log_2_) at 8 wpv. The chimeric bivalent 5 + 3 vaccinated mice showed a significant increase (*p* value < 0.001) of nAbs against MERS-CoV reaching to 1:160 (7.3 log_2_) at week 8 compared to the control group, as demonstrated in Figure 4. The control-PBS and inactivated WT 5 + 3 groups showed no nAbs in their sera against MERS-CoV throughout the duration of experiment.

### 2.4. Challenge Infection with Wild Type H1N1pdm09

To examine the protection efficiency of the vaccinated mice against H1N1pdm09 viral infection, H1N1pdm09 wild type was inoculated into BALB/c mice of PBS control, chimeric bivalent 5 + 3, WT5 + 3 vaccinated groups in our study. The recombinant chimeric bivalent 5 + 3 virus and positive control inactivated WT 5 + 3 showed no body weight loss till 14 days pi when compared with the control group. 

**Figure 5:**
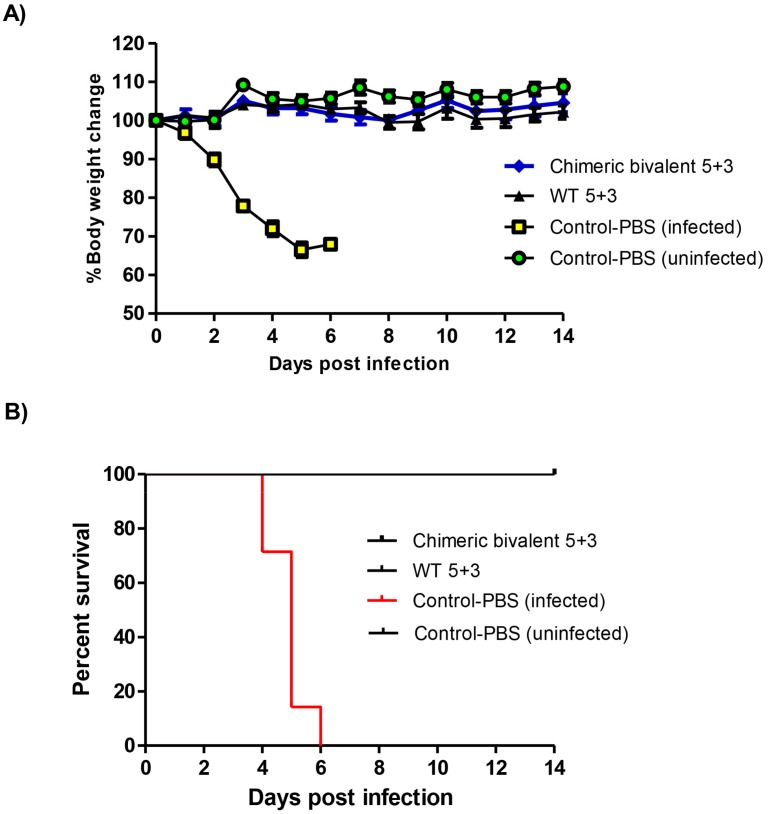
Body weight change and survival curve of BALB/c mice (seven mice/group) infected with 10^5.5^ TCID_50_ dose of H1N1pdm2009 wild type virus intra-nasally. (**A**) The loss in body weight recorded up to two weeks post infection (wpi) and (**B**) survival percent at indicated time points (up to 2 wpi). Mice were euthanized when they lost ≥30% of their original body weight.

All mice infected with H1N1pdm09 wild type in the control group had more than 30% decrease in weight from day 4 to 6 days pi leading to euthanasia (Figure 5A). The mortality rate in the control mice started at 4 days pi with 29% and increased gradually up to 85% at 5 days pi and 100% at 6 days pi (Figure 5B). These results pointed out that vaccination with either of the H1N1 viruses protected all mice from influenza-induced mortality.

## 3. Discussion

The MERS-CoV and pandemic influenza H1N1 viruses are among the most important emerging infectious diseases and represent serious public health challenges. The development of an effective bivalent vaccine against influenza and MERS-CoV infections is urgently required especially in the areas where both viruses are co-circulating. Thus, designing novel strategies to prevent MERS-CoV and influenza infections is in demand. A previous study showed that aa 736–761 of the MERS-CoV spike epitope elicit robust neutralizing activities and block viral infection [15]. Herein, we developed a novel recombinant bivalent vaccine against H1N1pdm09 and MERS-CoV using rescued influenza virus carrying the short immunogenic peptide 736–761 of the S protein in the NA gene. The short peptide of MERS-CoV was inserted at position 48 aa in the NA-stalk domain of influenza A/H1N1. This unique site was selected based on the distinct natural deletion in the corresponding neuraminidase subtype (N1) of H5N1 isolates in Egypt since 2006 [16,17]. For safety reasons, we preferred to test the NA of H1N1 instead of H5N1 as a shuttle for the MERS-CoV spike immunogenic epitope.

Reverse genetics was used for generation of recombinant influenza virus since it allows manipulation of influenza virus genome by addition, change, or removal of genetic determinants responsible for virus virulence. The short immunogenic peptide was inserted in the stalk region of NA from H1N1pdm09 using recombinant ligation. Influenza as a viral vector system for MERS-CoV has potential advantages including the ability for large-scale production of engineered virus in embryonated chicken eggs and stimulation of mucosal and systemic immune responses [18]. In our study, we used inactivated influenza vaccination due to its safety measure in comparison with influenza live attenuated vaccine (ILAV) which causes influenza like-illness (headache, runny nose, sore throat, and cough) and ILAV was limited in pregnant and immune-compromised patients [19]. 

The H1N1pdm09 vaccine virus grew poorly in embryonated chicken eggs compared with growth of previous seasonal H1N1 isolates [20], resulting in manufacturers struggling to meet demand. To overcome the limitation of replication of the rescued virus, in addition to the HA, and the modified NA, the PB1 segment of H1N1pdm09 strain was used to generate chimeric bivalent 5 + 3 candidate vaccine strain [21]. The developed vaccine seed virus replicated well without any associated adaptive mutations. Previous studies indicated that the insertion of reporter genes into the influenza viral NS, M, or NA-encoding segment was successful [22,23]. The inserted reporter genes in influenza viral genome were associated with inconstant expression levels and genome instability to dispose the foreign insertion. Our strategy of insertion of the short immunogenic peptide after 48 aa in the NA-coding region improved virus replication. 

The novel chimeric bivalent 5 + 3 vaccinated mice showed that a significant increase of nAbs against MERS-CoV occurs at week 8. Mice vaccinated with the chimeric bivalent virus were protected from mortality and body weight loss at 2 weeks of challenge with wild type H1N1pdm09 virus. At week 4, an apparent decline in antibody levels of specific immune response was observed. This is likely related to a traditional phenomenon, namely self-limitation or resolution, in which the neutralizing antibodies titer is decreased following the booster immunization as a response to the administrated antigens [24,25]. Several studies showed that the protective efficacy of MERS-CoV vaccines positively correlates with the evoked neutralizing antibody titers in the serum of vaccinated animals [26,27]. These results are in accordance with the results of other viral platforms chimeric viruses carrying spike protein which provided nAbs against MERS-CoV [9,28].

In summary, we demonstrated a new platform for generating replication-competent recombinant influenza chimeric bivalent 5 + 3 virus containing an immunogenic short peptide of MERS-CoV in the NA gene using reverse genetics technology. The rescued chimeric bivalent influenza virus could replicate well and was propagated for multiple passages in embryonated eggs. The novel recombinant bivalent IAV provided nAbs against MERS-CoV and HAI antibodies against H1N1pdm09 influenza viruses in vaccinated mice. Future studies for evaluation of this candidate vaccine for protective capacity in a murine-challenge model and non-human primates as a first step before human trails to generate a safe and well replicating candidate strain for human vaccines are recommended.

## 4. Materials and Methods 

### 4.1. Viruses

Influenza A virus A/California/04/2009 (H1N1, H1N1pdm09) and MERS-CoV/Camel/Egypt/HKU-NRCE205/2013 was obtained from Center of Scientific Excellence for Influenza Viruses (CSEIV), National Research Centre (NRC), Egypt. The influenza H1N1pdm2009 virus was propagated in Madin-Darby Canine Kidney (MDCK-II) cells, while the MERS-CoV strain was grown in African green monkey kidney cells (Vero-E6) cells. 

### 4.2. Construction of pHW-NAH1N1pdm09-MERS-CoV

We inserted a short immunogenic (25 aa) peptide ^736^CALPDTPSTLTPRSVRSVPGEMRLA^761^ from spike glycoprotein of MERS-CoV into the NA gene of H1N1pdm09 virus as illustrated in Figure 1A. To construct the chimeric pHW-NAH1N1pdm09-MERS-CoV plasmid, two PCR fragments were amplified using Ba-NA-1F: TATTGGTCTCAGGGAGCAAAAGCAGGAGT; Ba-NA-pdm09-peptide-R1: ATATGGTCTCACTGCGAGGTGTGAGAGTACTAGGTGTGTCAGG AAGA-GCACATGTTTCAATCTGATTTTGATT for fragment 1, and Ba-NA-pdm09-peptide-F2: ATTGGTCTCAGCAGTGTGCGCTCTGTTCCAGGTGAAATGCGCTTGGCATGCAATCAAAGCGTCATTACTTATG and Ba-NA-1413R: ATATGGTCTCGTATTAGT-AGAAACAAGGAGTTTTTT for fragment 2. Using Phusion Master Mix (Thermo, Waltham, MA, USA), in a total reaction 50 µL: 25 µL master mix, 3 µL each forward and reverse primers for each fragment, ddH2O 18 µL and template DNA (pHW-NAH1N1pdm09) 1 µL were mixed. Cycler conditions were 95 °C for 1 min followed by three steps (95 °C for 10 s, 58 °C for 30 s, and 72 °C for 2 min) for 40 cycles then final extension at 72 °C for 10 min. The two fragments were loaded in 1% agarose electrophoresis then bands were purified using QIAquick gel purification kit (Qiagen, Hilden, Germany). After purification, the two fragments digested with Bsa I (NEB, Ipswich, MA, USA). Ligation of the two fragments and BsmBI linearized pHW2000 [29] was done using T4 DNA ligase (Promega, Madison, WI, USA) overnight at 4 °C. Transformation using Top 10 competent cells was performed according to instructions. Colonies were selected for mini-prep. The purified construct was prepared for sequencing at Macrogen facility (Macrogen, Seoul, South Korea).

The HA and PB1 segments of A/California/04/2009 (H1N1) were amplified, digested using BsmBI (NEB, Ipswich, MA, USA), and ligated into linearized pHW2000 as previously described [29], to generate pHW-HAH1N1pdm09 and pHW-PB1H1N1pdm09. The plasmids carrying the eight gene segments of the high-yield A/PR8/8/1934 (H1N1) virus were supplied from St Jude Children’s Research Hospital, USA [29].

### 4.3. Generation of Recombinant Viruses by Reverse Genetics

Using the eight-plasmid reverse genetics system, recombinant rgH1N1pdm09 (WT 5 + 3), and rgH1N1-MERS-CoV (chimeric bivalent 5 + 3) viruses were generated using (pHW-PB2PR8, pHW-PB1H1N1pdm09, pHW-PAPR8, pHW-HAH1N1pdm09, pHW-NPPR8, pHW-NAH1N1pdm09, pHW-MPR8, and pHW-NSPR8) and (pHW-PB2PR8, pHW-PB1H1N1pdm09, pHW-PAPR8, pHW-HAH1N1pdm09, pHW-NPPR8, pHW-NAH1N1pdm09-MERS-CoV, pHW-MPR8, and pHW-NSPR8), respectively, as described previously [29]. Briefly, the day before transfection, 293T/MDCK-II cells were co-cultured in a ratio of 3/1 then incubated at 37 °C, 5% CO_2_. On the day of transfection, 8 μg of the eight desired DNA plasmids (1 μg per plasmid) was mixed with Trans-IT2020 (Mirus, Madison, WI, USA) (2 μL per 1 μg plasmid) and 184 µL of Opti-MEM reduced serum medium (Invitrogen, Carlsbad, CA, USA) and incubated for 45 min at room temperature. Afterwards, a volume of 800 μL of Opti-MEM medium was added to the mixture. The mixture was added drop wise to 293T/MDCK-II cells then incubated at 37 °C, 5% CO_2_ for 6 h. The transfection mixture was replaced with 1 mL Opti-MEM containing 1% antibiotic-antimycotic mixture, and 0.4% bovine serum albumin, and the cells were incubated overnight at 37 °C,5 % CO_2_. Subsequently, an additional 1 mL Opti-MEM containing Pen/Strep, 0.2% bovine serum albumin (BSA), and 2 μg L-1-Tosylamide-2-phenylethyl chloromethyl ketone (TPCK)-treated trypsin (Sigma-Aldrich, St. Louis, MI, USA) was added and the cells were further incubated for 48 h. The culture supernatants were harvested and clarified by low-speed centrifugation. Then, we injected the supernatant into the 10-day-old specific-pathogen-free (SPF) embryonated chicken eggs and MDCK-II cells. Standard hemagglutination test (HA) was used to detect the recombinant viruses in the allantoic fluid of embryonated chicken eggs and MDCK-II cells harvest. Recombinant viruses were harvested and propagated in SPF embryonated chicken eggs for three passages. The NA genes of the generated rescued viruses were amplified and sequenced to test stability of the inserted short peptide. 

### 4.4. Growth Kinetic for the Novel Recombinant Virus

MDCK-II cells were cultured in 6-well plates overnight to be confluent monolayers. Chimeric bivalent 5 + 3 and WT 5 + 3 viruses were infected with multiplicity of infection (MOI = 0.001) for 1 h at 37 °C in 5% CO_2_ incubator. The inoculum was removed and washed with 1× PBS then, a total amount of 2 mL (DMEM infection media) with 1 µg/mL TPCK treated trypsin was added to the cells. The supernatant of infected cells was harvested at specific time points and titrated in MDCK-II cells using 50% tissue culture infectious dose (TCID_50_) according to Reed and Muench protocol [30].

### 4.5. Preparation of Inactivated Vaccine

The novel rescued virus chimeric bivalent 5 + 3 and WT 5 + 3 were propagated in 10-day-old SPF embryonated chicken eggs. MERS-CoV was grown in Vero-E6 cells. The collected harvest for each virus was clear up from cell debris by centrifugation at 800× *g* for 5 min. Harvested viruses were inactivated using 0.1% formaldehyde and mixed well for 24 h at 4 °C. Inactivated viral particles were pelleted by careful layering of 30 mL of inactivated virus onto 12 mL of 20% sucrose in a centrifuge tube and then centrifuged in ultracentrifuge Sorvall MTX 150 (Thermo, Waltham, MA, USA) at 28,000 rpm at 4 °C for 2 h. The pellets were suspended in 500 µL 1× PBS and virus titers were measured by HA assay. Total Protein was measured using NanoDrop 2000 (Thermo Fisher Scientific, Waltham, MA, USA). Equal protein content of each inactivated virus was mixed with alum adjuvant 1:1 (v/v) for 30 min then these suspensions were used for immunization.

### 4.6. Mice Immunization and Serological Assays

Female (6–8 weeks-old) BALB/c mice were obtained from the animal house at NRC, Egypt. Mice were divided into four groups (seven mice/group). Three groups of mice were intramuscularly immunized with the three forms of inactivated chimeric bivalent 5 + 3, WT 5 + 3, and MERS-CoV vaccines. The remaining group served as a negative control and injected with sterile PBS. All animals received booster doses at 3 wpv. Sera were collected at 0, 2, 4, 6, and 8 wpv. The collected sera were tested for antibodies against H1N1pdm09 and MERS-CoV viruses using HAI [31] and PRNT_50_ in Vero-E6 cells. 

### 4.7. Plaque Reduction Neutralization Test (PRNT) 

PRNT assay was performed, as previously described [31], to determine the neutralizing capacity of elicited antibodies in sera from immunized BALB/C mice against MERS-CoV. Briefly, collected sera were firstly heated at 56 °C/30 min in water bath for inactivation. Sera were then two-fold serially diluted in 40 µL of DMEM/2% FBS (diluted from 1:20 to 1:160). To each sera dilution, an equal volume/amount of the plaque forming unit in 40 µL DMEM/2% FBS was supplied. After 1 h co-incubation of the serum/virus mixtures, 50 µL of each mixture were dispensed individually into 12-well tissue culture plates containing confluent monolayers of Vero-E6 cell. The cell monolayers were incubated together with the serum/virus mixtures at 37 °C for 1 h to allow virus adsorption. The infected Vero monolayers were washed with 1× PBS and supplied with agarose overlay containing 1× MEM media, 1% agarose, 1% Penicillin/Streptomycin (Pen/Strep), and then left to solidify. The plates were incubated until the formation of visible viral plaques (72 h). Cell monolayers were fixed with 3.4% formaldehyde solution for 1 h and stained with 1% crystal violet solution for 30 min at RT. Eventually, the plates were washed with water to visualize the plaques and the percent (%) of inhibition is calculated as following:

% of plaque reduction = (virus control plaques count − sample plaques count)/(virus control plaques count) × 100.

The PRNT_50_ is defined as the reciprocal of the antibody dilution required to reduce the number of MERS-CoV plaques in Vero-E6 cells by 50%, relative to the control wells.

### 4.8. Challenge Infection

Eight weeks post vaccination, immunized mice with chimeric bivalent 5 + 3, and WT 5 + 3 inactivated vaccines were anesthetized by injection intraperitoneal with ketamine with doses adjusted to their body weight (2 μg/g BWt). Influenza A virus wild type A/California/04/2009 (H1N1) was administered intranasally with infectious dose 10^5.5^ TCID_50_ to all immunized and control PBS groups. Mice showing a weight loss more than 30% of the initial body weight were euthanized and documented as dead. Body weight was monitored daily for 2 wpi. 

### 4.9. Ethics Statement and Biosafety

The animal trial in our study was conducted in accordance with the guidelines of the Egyptian animal welfare regulations and legislation. The ethics committee of the NRC, Egypt, approved the animal trial with number (16–247) and the regulations of Animal Welfare Assurance with identification number (A5939–01). All experiments were performed in a biosafety level 2 (BSL2) containment laboratory approved for such use by the local authorities (NRC, Egypt). For MERS-CoV neutralization assays, Class III Biological Safety Cabinet SEA-III BSC (Germfree, Ormond Beach, FL, USA) was used.

## References

[1] Zaki A.M., van Boheemen S., Bestebroer T.M., Osterhaus A.D., Fouchier R.A. (2012). Isolation of a novel coronavirus from a man with pneumonia in Saudi Arabia. N. Engl. J. Med..

[2] Gardner E.G., Kelton D., Poljak Z., Van Kerkhove M., von Dobschuetz S., Greer A.L. (2019). A case-crossover analysis of the impact of weather on primary cases of middle east respiratory syndrome. BMC Infect. Dis..

[3] Kim E., Okada K., Kenniston T., Raj V.S., AlHajri M.M., Farag E.A., AlHajri F., Osterhaus A.D., Haagmans B.L., Gambotto A. (2014). Immunogenicity of an adenoviral-based middle east respiratory syndrome coronavirus vaccine in balb/c mice. Vaccine.

[4] Guo X., Deng Y., Chen H., Lan J., Wang W., Zou X., Hung T., Lu Z., Tan W. (2015). Systemic and mucosal immunity in mice elicited by a single immunization with human adenovirus type 5 or 41 vector-based vaccines carrying the spike protein of middle east respiratory syndrome coronavirus. Immunology.

[5] Shehata M.M., Gomaa M.R., Ali M.A., Kayali G. (2016). Middle east respiratory syndrome coronavirus: A comprehensive review. Front. Med..

[6] Volz A., Kupke A., Song F., Jany S., Fux R., Shams-Eldin H., Schmidt J., Becker C., Eickmann M., Becker S. (2015). Protective efficacy of recombinant modified vaccinia virus ankara (mva) delivering middle east respiratory syndrome coronavirus spike glycoprotein. J. Virol..

[7] Song F., Fux R., Provacia L.B., Volz A., Eickmann M., Becker S., Osterhaus A.D., Haagmans B.L., Sutter G. (2013). Middle east respiratory syndrome coronavirus spike protein delivered by modified vaccinia virus ankara efficiently induces virus-neutralizing antibodies. J. Virol..

[8] Zhao J., Li K., Wohlford-Lenane C., Agnihothram S.S., Fett C., Zhao J., Gale M.J., Baric R.S., Enjuanes L., Gallagher T. (2014). Rapid generation of a mouse model for middle east respiratory syndrome. Proc. Natl. Acad. Sci. USA.

[9] Liu R., Wang J., Shao Y., Wang X., Zhang H., Shuai L., Ge J., Wen Z., Bu Z. (2018). A recombinant vsv-vectored mers-cov vaccine induces neutralizing antibody and t cell responses in rhesus monkeys after single dose immunization. Antivir. Res..

[10] Hause B.M., Collin E.A., Liu R., Huang B., Sheng Z., Lu W., Wang D., Nelson E.A., Li F. (2014). Characterization of a novel influenza virus in cattle and swine: Proposal for a new genus in the orthomyxoviridae family. MBio.

[11] Shaw M.L., Palese P. (2013). Orthomyxoviridae.

[12] Mostafa A., Abdelwhab E.M., Mettenleiter T.C., Pleschka S. (2018). Zoonotic potential of influenza a viruses: A comprehensive overview. Viruses.

[13] Hamilton J.R., Vijayakumar G., Palese P. (2018). A recombinant antibody-expressing influenza virus delays tumor growth in a mouse model. Cell Rep..

[14] Kotomina T., Korenkov D., Matyushenko V., Prokopenko P., Rudenko L., Isakova-Sivak I. (2018). Live attenuated influenza vaccine viral vector induces functional cytotoxic t-cell immune response against foreign cd8+ t-cell epitopes inserted into na and ns1 genes using the 2a self-cleavage site. Hum. Vaccines Immunother..

[15] Yang Y., Deng Y., Wen B., Wang H., Meng X., Lan J., Gao G.F., Tan W. (2014). The amino acids 736-761 of the mers-cov spike protein induce neutralizing antibodies: Implications for the development of vaccines and antiviral agents. Viral Immunol..

[16] El-Shesheny R., Kandeil A., Bagato O., Maatouq A.M., Moatasim Y., Rubrum A., Song M.S., Webby R.J., Ali M.A., Kayali G. (2014). Molecular characterization of avian influenza h5n1 virus in egypt and the emergence of a novel endemic subclade. J. Gen. Virol..

[17] El-Shesheny R., Kayali G., Kandeil A., Cai Z., Barakat A.B., Ghanim H., Ali M.A. (2012). Antigenic diversity and cross-reactivity of avian influenza h5n1 viruses in egypt between 2006 and 2011. J. Gen. Virol..

[18] Li J., Arevalo M.T., Zeng M. (2013). Engineering influenza viral vectors. Bioengineered.

[19] Grohskopf L.A., Alyanak E., Broder K.R., Walter E.B., Fry A.M., Jernigan D.B. (2019). Prevention and control of seasonal influenza with vaccines: Recommendations of the advisory committee on immunization practices—United States, 2019–2020 influenza season. MMWR Recomm. Rep. Morb. Mortal. Wkl. Rep. Recomm. Rep..

[20] Robertson J.S., Nicolson C., Harvey R., Johnson R., Major D., Guilfoyle K., Roseby S., Newman R., Collin R., Wallis C. (2011). The development of vaccine viruses against pandemic a(h1n1) influenza. Vaccine.

[21] Mostafa A., Kanrai P., Ziebuhr J., Pleschka S. (2016). The pb1 segment of an influenza a virus h1n1 2009pdm isolate enhances the replication efficiency of specific influenza vaccine strains in cell culture and embryonated eggs. J. Gen. Virol..

[22] Kittel C., Sereinig S., Ferko B., Stasakova J., Romanova J., Wolkerstorfer A., Katinger H., Egorov A. (2004). Rescue of influenza virus expressing gfp from the ns1 reading frame. Virology.

[23] Vieira Machado A., Naffakh N., Gerbaud S., van der Werf S., Escriou N. (2006). Recombinant influenza a viruses harboring optimized dicistronic na segment with an extended native 5′ terminal sequence: Induction of heterospecific b and t cell responses in mice. Virology.

[24] Laserson U., Vigneault F., Gadala-Maria D., Yaari G., Uduman M., Vander Heiden J.A., Kelton W., Taek Jung S., Liu Y., Laserson J. (2014). High-resolution antibody dynamics of vaccine-induced immune responses. Proc. Natl. Acad. Sci. USA.

[25] Rosendahl Huber S.K., Hendriks M., Jacobi R.H.J., van de Kassteele J., Mandersloot-Oskam J.C., van Boxtel R.A.J., Wensing A.M.J., Rots N.Y., Luytjes W., van Beek J. (2019). Immunogenicity of influenza vaccines: Evidence for differential effect of secondary vaccination on humoral and cellular immunity. Front. Immunol..

[26] Adney D.R., Wang L., van Doremalen N., Shi W., Zhang Y., Kong W.P., Miller M.R., Bushmaker T., Scott D., de Wit E. (2019). Efficacy of an adjuvanted middle east respiratory syndrome coronavirus spike protein vaccine in dromedary camels and alpacas. Viruses.

[27] Wang Y., Tai W., Yang J., Zhao G., Sun S., Tseng C.K., Jiang S., Zhou Y., Du L., Gao J. (2017). Receptor-binding domain of mers-cov with optimal immunogen dosage and immunization interval protects human transgenic mice from mers-cov infection. Hum. Vaccines Immunother..

[28] Wang C., Zheng X., Gai W., Wong G., Wang H., Jin H., Feng N., Zhao Y., Zhang W., Li N. (2017). Novel chimeric virus-like particles vaccine displaying mers-cov receptor-binding domain induce specific humoral and cellular immune response in mice. Antivir. Res..

[29] Hoffmann E., Neumann G., Kawaoka Y., Hobom G., Webster R.G. (2000). A DNA transfection system for generation of influenza a virus from eight plasmids. Proc. Natl. Acad. Sci. USA.

[30] Reed L.J., Muench H. (1938). A simple method of estimating fifty per cent endpoints. Am. J. Epidemiol..

[31] Shehata M.M., Mostafa A., Teubner L., Mahmoud S.H., Kandeil A., Elshesheny R., Frantz R., Pietra L., Pleschka S., Osman A. (2019). Bacterial outer membrane vesicles (omvs)-based dual vaccine for influenza a h1n1 virus and mers-cov. Vaccines.

